# The relationship between urban residents’ physical exercise attitudes and sports consumption demands: the mediating role of physical activity level

**DOI:** 10.3389/fpsyg.2024.1500150

**Published:** 2024-12-04

**Authors:** WeiSong Chen, Bowei Zhou, Bo Peng, Lin Li

**Affiliations:** ^1^Sports Training Academy, Chengdu Sport University, Chengdu, China; ^2^College of Physical Education, Southwestern University of Finance and Economics, Chengdu, China

**Keywords:** physical exercise attitudes, sports consumption demands, physical activity level, urban residents, Chengdu City

## Abstract

**Object:**

This study examines the differences and relationships among urban residents’ physical exercise attitudes, physical activity levels, and sports consumption demands, focusing on how physical activity mediates the relationship between exercise attitudes and consumption behaviors.

**Method:**

A survey was conducted among residents in Chengdu using validated questionnaires to measure physical exercise attitudes, physical activity levels, and sports consumption demands. Data analysis included independent samples t-tests and one-way ANOVA to assess differences across demographic variables, as well as correlation analysis and structural equation modeling to examine the path relationships among key variables.

**Results:**

Independent samples *t*-tests and ANOVA showed significant differences in sports consumption demands across age, education, and income groups (*p* < 0.05). Correlation analysis revealed significant positive relationships among physical exercise attitudes, physical activity levels, and sports consumption demands (*p* < 0.01). Structural equation modeling confirmed that physical activity levels partially mediated the relationship, with a direct effect size of 0.295 (*p* < 0.001) and an indirect effect size of 0.117 (*p* < 0.001), accounting for 28.4% of the total effect.

**Conclusion:**

The study highlights that positive exercise attitudes significantly impact sports consumption demands directly and through the mediating role of physical activity levels. Enhancing physical activity can amplify the effects of exercise attitudes on consumption, providing insights for promoting sports participation and consumption in urban settings.

## 1 Introduction

In contemporary society, the sports industry has emerged as a pivotal force in driving high-quality economic development (Li et al., 2022), with sports consumption serving as a core component that is gradually evolving into a new aspiration to meet the growing needs of people for a better life (Koronios et al., 2020). In recognition of this, the government has promulgated a series of policy documents aimed at promoting mass sports participation and consumption awareness, and fostering the high-quality development of the sports industry (Volf et al., 2022; Hughes et al., 2022). These policies not only provide a strong foundation for the vibrant growth of the sports industry (Hai, 2014), but also offer a solid policy basis for this study to explore the relationship between physical exercise attitudes and sports consumption demands.

However, the current situation of sports participation among Chinese residents is far from optimistic (Su et al., 2017; Gan and Jiang, 2022). The prevalence of sedentary lifestyles and other unhealthy behaviors has significantly hindered the growth of sports consumption demand (Dong and Liu, 2022; Dong et al., 2023). The inadequacy of sports participation has become one of the major constraints limiting the development of the sports industry (Li et al., 2023; Xing et al., 2020; Thibaut et al., 2020). Therefore, exploring the relationship between physical exercise attitudes and sports consumption demands holds significant importance in stimulating sports consumption and propelling the progress of the sports industry.

At the theoretical level, this study introduces the Theory of Planned Behavior (TPB) and the Technology Acceptance Model (TAM) to effectively link core variables such as physical exercise attitudes, physical activity level, and sports consumption demands. TPB emphasizes the crucial role of behavioral intention in predicting actual behavior (Armitage and Conner, 2001; Ajzen, 2011), with physical exercise attitudes and subjective norms being key factors influencing behavioral intention (La Barbera and Ajzen 2021; Rhodes et al., 2024; Phipps et al., 2021). Meanwhile, TAM approaches the issue from the perspective of consumer perception (Yau and Hsiao, 2022; Venkatesh et al., 2003), positing that perceived usefulness and perceived ease of use are significant determinants of consumer attitudes and behaviors (Rhodes et al., 2024; Ibrahim, 2013; Sanjida and Touhiduzzaman, 2022). These theories provide vital theoretical support and analytical frameworks for this study.

In summary, this study aims to investigate the relationship between physical exercise attitudes, physical activity level, and sports consumption demands. Through an in-depth examination of this topic, we aspire to offer insightful ideas and recommendations that can foster greater participation in sports activities, enhance sports consumption awareness, and promote the high-quality development of the sports industry.

## 2 Literature review and theoretical framework

### 2.1 Physical exercise attitude

In psychology, attitude refers to a stable psychological tendency held by an individual toward a specific object of inclination (person, idea, emotion, or event) (Fishbein and Ajzen, 1977), which implies the individual’s subjective evaluation and the resulting specific behavioral tendency (Rhodes et al., 2024). An attitude consists of three dimensions: cognition, affect, and behavioral intention (Bock et al., 2005). Cognition refers to the meaningful commentary made by an individual about the attitudinal object, including knowledge, understanding, doubt, and approval (Elizabeth, 2000). Affect concerns the emotional experience an individual has toward the attitudinal object, such as liking or disliking (Mcallister, 1995). Behavioral intention indicates the individual’s responsiveness and readiness to react toward the attitudinal object (Bock et al., 2005).

Regarding physical exercise attitude, scholars offer slightly varying interpretations. Some studies suggest that physical exercise attitude constitute a narrow component within the broader concept of sports attitude, referring specifically to an individual’s motivation to engage in physical exercise (Wen-Qian and Jian-Ping, 2006; Cui-Jin et al., 2012; Song, 2019). This motivation is seen as a relatively stable cognitive manifestation that can be acquired through learning (Cui-Jin et al., 2012; Wei-Qiang, 2017). Others define physical exercise attitude as the emotional and behavioral responses exhibited by individuals across different contexts (Qi-Feng, 2017), representing a comprehensive manifestation of cognition, affect, and behavioral intention toward physical exercise (Mei et al., 2019; Lee et al., 2021). Such attitudes are believed to exert a positive influence on subsequent exercise behaviors (Wen-Qian and Jian-Ping, 2006; Wei-Qiang, 2017; Sindik et al., 2022). Furthermore, physical exercise attitude also encompass specific exercise content, determining the types and forms of exercise an individual chooses to participate in and ultimately affecting the effectiveness of their physical training (Xu and Yu, 2013).

Some scholars have further subdivided attitude toward physical exercise into eight dimensions: behavioral attitude, goal attitude, behavioral cognition, behavioral habit, behavioral intention, emotional experience, sense of behavioral control, and subjective norm (Jaarsma et al., 2014).

In this study, physical exercise attitude is defined as the individual’s cognitive, affective, and behavioral intention specifically toward engaging in physical exercise. This definition focuses on attitudes that drive direct participation in physical activities, including motivation, emotional response, and readiness to engage in exercise. This study employs this dimensional framework—encompassing cognition, affect, and behavioral intention—to investigate how attitudes toward physical exercise influence sports consumption demand among urban residents.

### 2.2 Physical activity level

The term “Physical Activity Level” refers to the amount of physical activity engaged in by an individual (Xu, 2013). Physical activity level falls within the scope of sports behavior and is considered its primary manifestation (Piątkowska, 2012). To delve into the physical activity level, one must first clarify what is meant by “physical exercise,” also known as bodily exercise (Caspersen et al., 1985). It refers to physical activities aimed at improving health, enhancing physical fitness, regulating one’s spirit, and enriching cultural life (Surkalim et al., 2024). Studies have shown that sports behavior entails the conscious adoption of sports methods and means to achieve sporting goals (Kuczkowska, 2021). Exercise behavior mainly occurs during leisure time, with health as the primary objective, and involves bodily activities of certain intensity, frequency, and duration (Wang, 2015). Some define it as leisure-time physical activity, which is a subset of physical activity (Li, 2012). Studies have shown that sports behavior is a conscious adoption of sports methods and means to achieve sports goals (Shenoi et al., 2022).

In this study, physical activity level is defined as the extent of involvement in sports-specific activities that are structured, planned, and undertaken with the goal of improving or maintaining physical health, such as recreational exercise and fitness activities. This definition intentionally excludes physical activities related to daily routines, such as household chores, gardening, work, or transport. The measurement tool applied in this research (i.e., the Physical Activity Rating Scale) assesses the level of engagement in sporting activities, with a focus on intensity, frequency, and duration, to ensure that the assessment aligns specifically with sports-related physical activity rather than general daily movements.

### 2.3 Sports consumption demand

Consumption demand refers to the desire and willingness of consumers to acquire various consumption materials to fulfill their needs for survival, enjoyment, and development (Bronsard, 1981; Liu, 2000). Therefore, demand for sports consumption represents the degree of consumers’ desire for various sports products (Chen et al., 2008).

Sports consumption demand can be understood as an inevitable reflection of the organism’s demand for sports consumption when people have basically met their physiological and safety needs, in pursuit of health, entertainment, social interaction, respect, and other needs (Liu, 2000; Pang and Li, 2016). It is a product of social productive forces developing to a certain stage and an indispensable component of the consumption structure of modern people’s lives (Liu et al., 2004). From the perspective of the objects of sports consumers’ demand, it can be divided into two major categories: material consumption of sports and spiritual consumption of sports (Chen et al., 2008). Material consumption demand mainly refers to people’s demand for various physical sports products (such as sportswear, fitness equipment, etc.) (Chen et al., 2008). Spiritual consumption demand mainly refers to people’s demand for watching sports competitions, participating in sports activities, training, enjoying sports services, etc., to meet physiological and spiritual needs (Chen et al., 2008).

Some studies have pointed out the general laws of sports consumption behavior and believe that sports demand is the starting point for sports consumption behavior (Liu, 2000; Pang and Li, 2016; Shasha, 2023). At the same time, research indicates that sports consumption demand is built on individuals’ basic physiological and safety needs, arising from the need for health, entertainment, social interaction, etc., and is an inevitable product of the continuous development of social productive forces (Weimar and Breuer, 2022; Guo and Chen, 2022).

Sports consumption demand encompasses a wealth of content, and its classification should be hierarchical, specifically including participation demand, tangible demand, information demand, and ornamental demand (Chen et al., 2008). The attributes it possesses transition continuously and hierarchically from material to spiritual (Chen et al., 2010).

In this study, sports consumption demand is defined as the multifaceted desire and willingness of individuals to engage in both material and spiritual aspects of sports. This encompasses not only the direct purchase of sports products and services but also the broader consumption of sports experiences, such as participating in or spectating sports activities, as well as engaging with sports-related content for entertainment and social interaction. This study aims to capture both active involvement (e.g., direct participation) and passive engagement (e.g., spectating and consuming sports media), providing a comprehensive perspective on the ways urban residents interact with sports as consumers.

### 2.4 Physical exercise attitude and sports consumption demand

Investigations into the dynamics of physical exercise attitude and sports consumption demand reveal an evolving relationship tied to the various stages of exercise engagement (Xu, 2013). Initial studies chart a clear trajectory of rising sports consumption as individuals progress from contemplation (pre-intention stage) to active participation (action stage), with a noted dip during the habituation (maintenance stage) (Chen et al., 2012).

In the context of university students, a nuanced pattern emerges, illustrating an escalation in sports-related spending correlating with the advancement through exercise stages (Chen, 2020). Early stages see modest expenditure, under 100 yuan monthly, which notably increases to between 100 and 300 yuan as students reach the maintenance phase of their exercise routines (Jiao et al., 2018).

In addition, investigations in certain regions of China have shed light on how attitudes toward physical activity influence sports consumption behaviors. Findings suggest that students with a positive disposition toward regular physical exercise tend to have higher sports consumption, illustrating the significant connection between physical exercise attitudes and spending behaviors on sports (Cui-Jin et al., 2012; Wei-Qiang, 2017; Fan, 2012).

Further analysis extends to the adolescent demographic, dissecting sports consumption into three distinct needs: intrinsic (self-purpose), supportive (auxiliary), and functional (instrumental) consumption. The study found an important link between daily physical activity attitudes and these different consumption categories, underscoring a strong correlation even after adjusting for demographic effects (Chen, 2020; Yang et al., 2020). Positive attitudes toward extracurricular physical activity are clearly aligned with these identified sports consumption demand, highlighting the comprehensive interplay between physical exercise attitudes and sports consumption demand at different life stages and activities (Chen, 2020; Mao, 2003).

### 2.5 Physical exercise attitude and physical activity level

When exploring the relationship between physical exercise attitude and physical activity level, studies have found limitations in traditional planned behavior theory, as it ignores the potential impact of emotions, goal attitudes, and habits on exercise intentions and behaviors (Martínez-de-Quel et al., 2021). To address this gap, researchers have proposed a more comprehensive “Exercise-Behavior Nine-Factor Model” and designed a corresponding physical exercise attitude scale (Hagger and Hamilton, 2020), which has been widely used in China to assess the exercise attitudes of groups such as adolescents and college students.

Further empirical research has revealed a certain correlation between physical exercise attitude and physical activity levels. In a study targeting college students, researchers used the physical exercise attitude scale and the physical activity rating scale, finding that apart from a significant negative correlation between subjective norms and exercise volume, the other seven dimensions of exercise attitude all showed a weak positive correlation with exercise volume (Zhou and Zhou, 2009). Another study on urban residents indicated that physical exercise attitude, subjective norms, and cognitive behavior control jointly influenced their physical activity levels (Frmel et al., 2020). Additional research has explored the predictive effect of physical exercise attitude on the exercise behavior activity levels of college students in specific regions (such as Heilongjiang Province), finding that physical exercise attitude had a good predictive effect on exercise intensity and frequency but a less satisfactory predictive effect on exercise duration (Liu and Li, 2011).

### 2.6 Physical activity level and sports consumption demand

The relationship between physical activity level and sports consumption demand has garnered attention through various studies, particularly focusing on university students in urban settings (Knaggs et al., 2022). Research conducted in metropolitan areas shows a clear positive correlation between the frequency and intensity of physical activities and the demand for sports-related products and services among students (Zhen-Long et al., 2009). This link underscores the direct impact that exercise behaviors have on sports consumption patterns.

Moreover, a study spanning a range of ages from schoolchildren to university students revealed that specific exercise routines significantly affect the diversity and type of sports consumption, pointing to varied preferences based on individual characteristics and interests (Chen, 2020).

However, despite these insights, there remains a gap in research specifically targeting the urban resident population (Fan, 2012; Wirnitzer et al., 2023). This gap presents an opportunity for further exploration, especially considering the changing socio-economic conditions, rising disposable incomes, and the growing proliferation of fitness initiatives (Fang et al., 2022). Addressing these factors in future studies could offer deeper understanding into consumer behaviors and demands within the ever-evolving sports market.

Although there are many researches on the pair-to-pair correlation between physical exercise attitude, physical activity level and sports consumption demand, no studies have been found on the relationship between the three. Based on this, this study includes physical exercise attitude, physical activity level and sports consumption demand at the same time, and discusses the relationship among the three.

According to the relevant studies reviewed in literature, there is a pin-wise correlation between the physical exercise attitude, physical activity level and sports consumption demand, but the relevant studies are also controversial, that is, the specific correlation between the three dimensions may not be significant. At the same time, the research on the physical exercise attitude, physical activity level and sports consumption demand is mostly concentrated on the study of college students, but the research is relatively scarce in the urban residents in our country. Therefore, this study takes Chengdu City as an example to explore the relationship among urban residents’ physical exercise attitude, physical activity level and sports consumption demand. Based on the results of the above literature review, the following hypotheses are proposed:

*H1*: There are significant differences in the sports consumption demand of Chengdu City residents with different demographic variables.

*H2*: There is a significant positive correlation between the physical exercise attitude, physical activity level, and sports consumption demand of residents in Chengdu City.

*H3*: The physical activity level of Chengdu residents mediates the relationship between physical exercise attitude and sports consumption demand.

### 2.7 Current study

In the context of the burgeoning sports industry and the evolving landscape of urban lifestyles, there’s a pressing need to understand the dynamics between urban residents’ physical exercise attitude, their actual engagement in physical activities, and the resultant sports consumption demands. Previous research has often explored these elements in isolation, examining either the relationship between physical exercise attitude and sports consumption demand or the impact of physical activity levels on consumption patterns. Yet, the interconnectedness of these factors and their collective influence on sports consumption demand remains underexplored, especially within urban Chinese settings such as Chengdu City.

The present research seeks to fill this gap by proposing an integrative model that examines the relationships among physical exercise attitudes, physical activity levels, and sports consumption demands within an urban context. Specifically, the study aims to:

1. Assess the direct relationship between urban residents’ physical exercise attitudes and their sports consumption demands.
2. Explore the extent to which physical activity levels mediate the relationship between physical exercise attitudes and sports consumption demands.
3. Investigate the compounded effects of physical exercise attitudes and physical activity levels on the variety and intensity of sports consumption among urban residents.

This research makes several significant contributions to both theory and practice:

1. Theoretical Expansion: By integrating the Theory of Planned Behavior (TPB) and the Technology Acceptance Model (TAM), this study extends existing theoretical frameworks to the context of sports consumption. This novel approach allows for a comprehensive understanding of how attitudes toward physical exercise influence consumption behaviors through the mediating role of physical activity levels.
2. Empirical Insights: Offering empirical evidence from Chengdu City, the study enriches the body of knowledge on sports consumption behaviors in urban China. It provides nuanced insights into how exercise attitudes and engagement levels influence sports consumption, addressing a significant gap in the current literature.
3. Policy and Marketing Implications: The findings are poised to inform policymakers and marketers about the underlying motivations of sports consumption. By understanding the role of exercise attitudes and activity levels, strategies can be devised to promote healthier lifestyles and boost sports consumption in urban settings.
4. Sociocultural Perspective: Given the unique sociocultural dynamics of urban Chinese populations, this research contributes to the global discourse on sports consumption by highlighting the influence of local environmental and societal factors.

## 3 Methods

### 3.1 Participants

This study utilized three questionnaires, comprising a total of 83 items alongside demographic data collection, resulting in approximately 90 survey items. Following the 1:10 ratio principle commonly applied in social science surveys (i.e., 10 times the number of questionnaire items), the minimum required sample size was set at 900 respondents. To account for potential invalid responses, a total of 1,500 residents were initially recruited.

The inclusion criteria ensured that all participants were adults aged 18 years or older. Participation was voluntary and anonymous, with informed consent obtained verbally from all respondents prior to the survey. Exclusion criteria for the data included: (1) substantial omissions in questionnaire responses; (2) random answering patterns (e.g., repetitive or illogical sequences such as “1” shape or “Z” shape patterns); and (3) height and weight values significantly deviating from normal ranges. For questionnaires with minor omissions or errors, missing values were handled through linear interpolation.

After excluding invalid questionnaires, a total of 1,227 valid responses were obtained. Among the 1,227 participants, the age distribution was as follows: 10.59% were aged 18–20, 36.92% were aged 21–30, 30.48% were aged 31–40, 17.11% were aged 41–50, and 4.89% were over 51 years old. The sample was balanced in terms of gender, with 50.04% male and 49.96% female participants. Educational backgrounds included 3.67% with junior high school education or below, 27.38% with high school/technical secondary/vocational/technical education, 26.89% with junior college degrees, 37.65% with bachelor’s degrees, and 4.40% with graduate degrees. Monthly income levels were reported as 1.71% below 1,500 RMB, 22.74% between 1,501 and 2,000 RMB, and 75.55% above 2,001 RMB. Sports consumption levels were 62.51% below 100 RMB, 23.47% between 101 and 300 RMB, 10.35% between 301 and 500 RMB, and 3.67% above 501 RMB.

### 3.2 Measurement tools

#### 3.2.1 Physical Exercise Attitude Scale

This study utilized the “Exercise Attitude Scale” developed by Mao (2003), to measure the physical exercise attitudes among urban residents of Chengdu City. The scale comprises 70 items across eight dimensions: behavioral attitude, goal attitude, behavioral cognition, behavioral habit, behavioral intention, emotional experience, sense of behavioral control, and subjective norms. Each item is rated on a 5-point scale ranging from “strongly disagree” to “strongly agree,” with scores from 1 to 5, respectively. A higher score indicates a more positive attitude toward exercise.

Except for the subjective norms dimension, which has a Cronbach’s *α* coefficient above 0.6, the remaining dimensions all have Cronbach’s *α* coefficients above 0.8, demonstrating good reliability. Factor loadings for the survey questions related to this construct (Physical exercise attitude) ranged from 0.656 to 0.823. The scale’s construct reliability (CR), which is higher than the evaluation cutoff limit of 0.60, was scored at 0.882. The AVE score of the scale was 0.637, over the assessment requirement of 0.50. This implies that the construct validity and discrimination of the scale are excellent. The scale’s goodness of fit test findings was as follows: the following GFI, AGFI, NFI, IFl, CFI, and RMSEA values indicate that the scale has a satisfactory goodness of fit: 0.971, 0.966, 0.901, 0.977, and 0.041.

#### 3.2.2 Physical Activity Level Scale

This study adopted the revised “Physical Activity Rating Scale (PARS-3)” by Liang (1994), to investigate the physical activity levels among urban residents of Chengdu City. The scale assesses the volume of physical activity among college students from three aspects: intensity of physical exercise, duration, and frequency, each divided into five levels. The intensity and duration of physical exercise are rated from 1 to 5, while the frequency of physical exercise is rated from 0 to 4. The test–retest reliability of this questionnaire is 0.82, indicating relatively high reliability. The formula for calculating physical activity level is: Physical Activity Level = Intensity of Physical Exercise × Duration of Physical Exercise × Frequency of Physical Exercise. The evaluation criteria for Physical Activity Levels are: ≤19 points for low volume, 20–42 points for moderate volume, and ≥43 points for high volume.

#### 3.2.3 Sports Consumption Demand Scale

This study employed the “Sports Consumption Demand Scale” developed by Chen et al. (2010), to survey the sports consumption demands of urban residents in Chengdu City. Using a Likert 5-point scale, the scale evaluates sports consumption demands through 10 items across four dimensions: participation demand, tangible demand, information demand, and ornamental demand.

All four dimensions of the scale have Cronbach’s *α* coefficients exceeding 0.7, showing good internal consistency reliability. Every survey question had a factor loading that varied from 0.649 to 0.823. The scale’s construct reliability (CR) rating was 0.857, above the evaluation cutoff point of 0.60. The AVE value of the scale was 0.586, over the 0.50 evaluation threshold. This implies that the discrimination and construct validity of the scale are quite strong. The following outcomes of the scale’s goodness of fit test were obtained: For the scale, the following values of GFI, AGFI, NFI, IFI, CFI, and RMSEA suggest a sufficient goodness of fit: 0.987, 0.975, 0.976, 0.989, and 0.023.

### 3.3 Procedures

The research employed a stratified cluster sampling method, randomly selecting Wuhou District, Qingyang District, Jinjiang District, Chenghua District, and Jinniu District from among the 12 districts and counties of Chengdu City for the survey, with 300 questionnaires distributed in each urban district. The distribution of questionnaires aimed to ensure a balanced representation of participants’ ages and genders as much as possible. All questionnaires were completed on-site and collected immediately.

Data processing and statistical analysis were conducted using SPSS 25.0, Stata 12.0, and Amos 23.0. Parametric tests (independent samples *t*-test and one-way ANOVA) were applied for comparing demographic variables. The relationships among Physical Exercise Attitude, Physical Activity Level, and Sports Consumption Demand were explored using Pearson correlation. Additionally, the study examined the mediating role of Physical Activity Level between Physical Exercise Attitude and Sports Consumption Demand. Model fit was evaluated using CMIN/DF, RMSEA, NFI, RFI, IFI, TLI, and CFI indicators, with acceptable fit criteria set according to standard thresholds. The significance level for all statistical tests was set at *α* = 0.05 (Hayes, 2017).

## 4 Results

### 4.1 Common method bias and multicollinearity test

To mitigate potential common method bias, this study employed anonymous questionnaires and varied scoring techniques. Common method bias was assessed using the Harman single-factor test (Zhou and Long, 2004). An unrotated factor analysis revealed 13 factors with eigenvalues exceeding 1, and the first factor accounted for 29.19% of the total variance, which falls below the critical threshold of 40%. Additionally, the Variance Inflation Factor (VIF) for each scale was found to be less than 5, indicating no issues of multicollinearity (Hair et al., 1998).

### 4.2 Differences in sports consumption demand and demographic variables

The survey results showed that the overall score for Sports Consumption Demand was 38.21 ± 7.79. Independent samples t-tests and one-way ANOVA analyses were conducted to examine differences across various demographic variables. Significant differences in Sports Consumption Demand were observed across different age groups, with residents aged 21–30 (39.19 ± 6.60) and 31–40 (38.87 ± 7.49) exhibiting higher demand compared to other age groups. Differences were also noted among residents with different educational levels, where those with a junior college education showed the highest demand (39.44 ± 7.89). Furthermore, significant differences in demand were found based on monthly consumption levels and monthly sports spending. Interestingly, residents with higher monthly sports spending demonstrated lower sports consumption demand. However, no significant difference was observed between male and female residents.

These findings provide insights into how sports consumption demand varies with demographic characteristics, supporting Hypothesis 1. Detailed results are presented in Table 1.

**Table 1:** Differences in sports consumption demand across demographic variables.

Demographic variable	Group	Mean ± SD	Statistical test result
Age	18–20	36.28 ± 8.37	*F* = 13.081, *p* < 0.01
	21–30	39.19 ± 6.60	
	31–40	38.87 ± 7.49	
	41–50	38.00 ± 8.38	
	>51	32.34 ± 9.87	
Education level	High school or below	36.64 ± 8.19	*F* = 9.208, *p* < 0.05
	Junior college	39.44 ± 7.89	
	Bachelor’s degree	38.48 ± 6.83	
	Graduate degree	35.89 ± 8.54	
Monthly consumption	<1,500 RMB	34.84 ± 7.38	*F* = 4.663, *p* < 0.05
	1,501–2000 RMB	37.48 ± 8.32	
	>2001 RMB	38.54 ± 7.54	
Monthly sports spending	<100 RMB	38.97 ± 7.26	*F* = 9.134, *p* < 0.05
	101–300 RMB	37.53 ± 8.06	
	301–500 RMB	36.69 ± 8.35	
	>501 RMB	34.11 ± 8.21	
Gender	Male	38.3388 ± 7.48152	*t* = 0.557, *p* > 0.05
	Female	38.1109 ± 8.07304	

### 4.3 Analysis of the relationship between physical exercise attitude, physical activity level, and sports consumption demand

Through Pearson correlation analysis, the relationships among physical exercise attitude, physical activity level, and sports consumption demand were explored (Table 2). The study found significant positive correlations between each pair of these three variables, supporting Hypothesis 2 of this research.

**Table 2:** Correlation analysis of physical exercise attitude, physical activity level, and sports consumption demand (*r*).

Variables	Physical exercise attitude	Physical activity level	Sports consumption demand
Physical exercise attitude	1		
Physical activity level	0.374**	1	
Sports consumption demand	0.348**	0.364**	1

Significant *p < 0.05, **p < 0.01, ***p < 0.001.

### 4.4 Fitting model

The study conducted a path analysis with residents’ physical exercise attitude as the independent variable, residents’ sports consumption demand as the dependent variable, and the physical activity level as the mediating variable. The results of the model fit tests showed that CMIN/DF = 2.724, RMSEA = 0.047, with NFI, RFI, IFI, TLI, and CFI values being 0.988, 0.985, 0.991, 0.989, and 0.991, respectively, indicating an overall good fit of the model (Table 3).

**Table 3:** Fitting indices.

Index	Value
CMIN/DF	2.724
NFI	0.988
RFI	0.985
IFI	0.991
TLI	0.989
CFI	0.991
RMSEA	0.047

### 4.5 Structural model

Maximum likelihood estimation was used to calculate the coefficients for each path, and the results (Table 4) showed that the coefficients for Paths 1, 2, and 3 were statistically significant (*p* < 0.001), indicating that the pathway from physical exercise attitude to residents’ sports consumption demand through the physical activity level is valid.

**Table 4:** Direct estimations.

Paths	Relationships	Standardized estimates	SE	C.R.	*P*
1	Physical exercise attitude → Physical activity level	0.409	0.066	8.455	<0.001
2	Physical exercise attitude → Sports consumption demand	0.295	0.068	5.618	<0.001
3	Physical activity level → Physical exercise attitude	0.287	0.049	5.537	<0.001

### 4.6 Mediation analysis

To ascertain the mediating influence of physical activity level on the relationship between physical exercise attitude and sports consumption demand, the present investigation utilized the Bootstrap approach within the AMOS software. The analysis executed 5,000 bootstrap samples applying both the Bias-corrected and Percentile methods.

Table 5 presents the mediation analysis examining the total, direct, and indirect effects of physical exercise attitude on sports consumption demand, with physical activity level serving as the mediator. The total effect was significant with an effect size of 0.412 (Boot SE = 0.044, *p* < 0.001), and 95% confidence intervals ranging from 0.319 to 0.495 in the bias-corrected CI and 0.321 to 0.496 in the percentile CI, accounting for the combined direct and indirect pathways. The direct effect of physical exercise attitude on sports consumption demand, not mediated by physical activity level, was also significant, with an effect size of 0.295 (Boot SE = 0.051, *p* < 0.001), and identical lower and upper bounds for both bias-corrected and percentile 95% CIs (0.193–0.390), constituting 71.60% of the total effect. The indirect effect through the mediator—physical activity level—had an effect size of 0.117 (Boot SE = 0.023, *p* < 0.001), with confidence intervals that did not include zero (bias-corrected CI: 0.077–0.170; percentile CI: 0.075–0.167), representing 28.40% of the total effect. These findings suggest that physical activity level is a significant mediator in the relationship between physical exercise attitude and sports consumption demand.

**Table 5:** Analysis of total effect, direct effect, and indirect effect.

	Effect	Boot SE	*P*	Bias-corrected 95% CI		Percentile 95% CI		Effect ratio (%)
				Lower	Upper	Lower	Upper	
Total effect	0.412	0.044	<0.001	0.319	0.495	0.321	0.496	
Direct effect	0.295	0.051	<0.001	0.193	0.390	0.193	0.390	71.60
Indirect effect of physical activity level	0.117	0.023	<0.001	0.077	0.170	0.075	0.167	28.40

**Figure 1:**
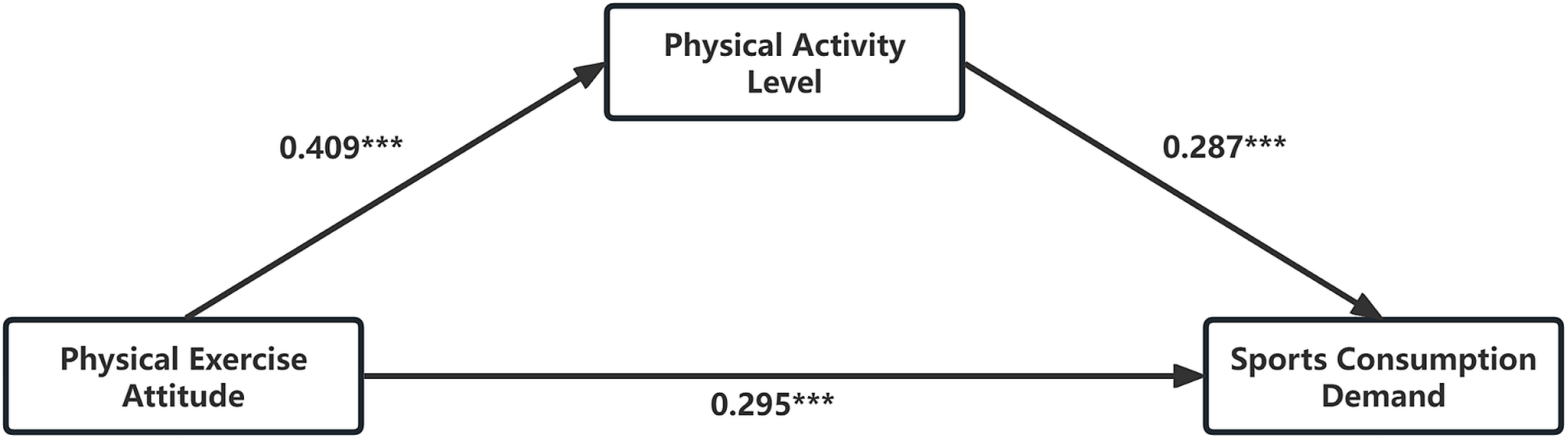
Mediating effect model. ***P < 0.001.

The model proposed in Figure 1 is thus supported as a partial mediation model, characterized by significant direct and indirect pathways that confirm the hypotheses 3 set forth in the study. The mediation role of physical activity level is statistically significant, and the model demonstrates substantial indirect effects, validating the study’s theoretical framework.

## 5 Discussion

The findings from this study’s survey, presenting a score of 38.21 ± 7.79 for sports consumption demand, provide empirical support for Hypothesis H1, which posits significant differences in sports consumption demand among Chengdu City residents based on various demographic variables. Independent samples *t*-tests and one-way ANOVA reinforced this hypothesis by revealing statistically significant differences across age groups, educational levels, and income brackets.

Age, as an influential demographic factor, displayed a marked variance in sports consumption demand (*F* = 13.081, *p* < 0.01), revealing that younger age cohorts, specifically those aged 21–30 and 31–40, had higher consumption demand. This finding correlates with the lifestyle and energy expenditure patterns of these age groups, often associated with heightened physical activity and thus a greater engagement in sports-related consumption (Pang and Li, 2016; Liu and Zhong, 2023). The reduced demand observed in the 41–50 age bracket and notably lower demand for those over 51 may reflect shifts in lifestyle, priorities, or physical capabilities that accompany aging (Xu and Yu, 2013; Liu, 2000), a trend well-documented in consumer behavior research. These age-related variations suggest that targeted marketing strategies could be particularly effective if tailored to the unique demands and lifestyle shifts of different age groups. For instance, younger individuals may respond to marketing that emphasizes social and dynamic aspects of sports, while older groups may benefit from promotions highlighting health maintenance and wellness.

Education level is another demographic determinant of consumption patterns, with this study demonstrating a significant effect on sports consumption demand (*F* = 9.208, *p* < 0.05). Residents with junior college education exhibited the highest demand, perhaps indicative of a more pronounced awareness or value placed on health and fitness, which could be attributed to educational curricula that emphasize these aspects (McGee and Adriana, 2014; Hua, 2022). Interestingly, those with graduate degrees showed the lowest demand, potentially due to time constraints or other commitments often associated with higher educational pursuits (McGee and Adriana, 2014; Kiani and Keivan, 2021). This education-based finding has implications for public health policies and educational programs. Promoting the benefits of sports participation within educational institutions, especially at advanced education levels, may encourage a culture of lifelong sports engagement, potentially offsetting the decreased demand observed among highly educated groups.

Monthly consumption levels also significantly differentiated sports consumption demand (*F* = 4.663, *p* < 0.05), underscoring the role of disposable income in the propensity to spend on sports. Residents with higher overall consumption levels likely have greater financial flexibility to allocate funds toward sports, affirming findings from economic theories on consumer spending (Xiaojuan and Yilu, 2020). In contrast, when isolating monthly sports consumption levels, a surprising inverse pattern emerged (*F* = 9.134, *p* < 0.05), where higher outlay on sports did not correspond to greater demand. This may suggest a saturation point in sports consumption or the presence of other factors such as time limitations or perceived value from spending that influences demand (Koronios et al., 2020; Hughes et al., 2022; Tang, 2023). These income-related insights highlight the importance of adaptive pricing strategies and product offerings tailored to different income brackets, which can help maximize engagement across varying economic backgrounds.

The absence of a significant gender difference (*t* = 0.557, *p* > 0.05) in sports consumption demand within Chengdu’s urban population may reflect a cultural context where sports engagement and related expenditures are becoming more gender-neutral, reflecting broader global trends toward gender equality in sports (Kateshumbwa, 2012; Uyar et al., 2022). This finding regarding gender neutrality in sports consumption is valuable for informing gender-inclusive marketing strategies and health initiatives that cater to both men and women without bias, reinforcing efforts toward equal access and engagement in sports activities.

These demographic insights bolster the understanding of sports consumption behaviors and are instrumental for strategizing in sports marketing and policymaking. The findings contribute to a nuanced portrayal of sports consumer demographics, where age, education, and income levels play crucial roles in shaping demand, offering a vantage point for future research and industry application.

The intricate interplay between physical exercise attitude, physical activity level, and sports consumption demand has been a subject of considerable interest in the domain of sports economics and psychology. The present study advances this scholarly dialog by providing empirical evidence of significant positive correlations between these variables, hence supporting Hypothesis 2.

The positive correlation between physical exercise attitude and physical activity level (0.374**, *p* < 0.01) aligns with the theoretical propositions of the Theory of Planned Behavior (Ajzen, 1991), which suggests that a positive attitude toward an activity is likely to result in increased participation. This correlation echoes the findings of previous research, which assert that attitudes toward exercise significantly predict the likelihood of engaging in physical activity (Armitage and Conner, 2001; Ajzen, 2011).

Furthermore, the relationship between physical exercise attitude and sports consumption demand (0.348**, *p* < 0.01) reinforces the concept that favorable perceptions and beliefs about exercise are closely linked to consumer spending on sports-related goods and services. This nexus can be traced to consumer behavior theory (Hansen, 1972), where attitudes have been identified as key predictors of consumption intentions (Glenn, 2021).

The positive correlation observed between physical activity level and sports consumption demand (0.364**, *p* < 0.01) offers empirical substantiation to the idea that individuals who are more active are also more likely to spend on sports. This is in line with findings from Zhen-Long et al. (2009), who identified that active engagement in physical activities tends to result in higher expenditure on sports commodities and services, likely due to the increased usage and, consequently, the need for sports-related products.

It is imperative to note that these correlations contribute to a more nuanced understanding of consumer behaviors within the sports market. For instance, while it is evident that attitudes and activity levels are positively correlated with sports consumption, there remains a complexity in these relationships that warrants further investigation. For example, the exact motivational drivers and the role of subjective norms in shaping these attitudes and behaviors may offer additional insights (Sanjida and Touhiduzzaman, 2022; Bock et al., 2005).

The mediation analysis undertaken in this study lends substantial support to the behavioral economics of sports consumption, dovetailing with the principles of the Theory of Planned Behavior (TPB) and the Technology Acceptance Model (TAM) to dissect the interplay between attitude, behavior, and economic outcomes (La Barbera and Ajzen 2021; Rhodes et al., 2024; Yau and Hsiao, 2022). Applying a Bootstrap methodology with AMOS software and conducting 5,000 bootstrap samples, this study substantiates the proposed mediating role of physical activity level among Chengdu’s urban dwellers.

The direct effect size of 0.295, accounting for 71.60% of the total effect, and the indirect effect size of 0.117, representing 28.40%, delineate the complexity of the relationship between attitude and consumption. This aligns with the theory within the framework of the Theory of Planned Behavior, which asserts that attitude significantly impacts an individual’s intentions and subsequent behaviors, including consumption practices (La Barbera and Ajzen 2021; Rhodes et al., 2024).

Notably, the indirect effect confirms that physical activity level is not merely a byproduct of positive attitudes toward exercise but a substantive mediator that can influence consumption demand. This finding echoes the research who posits that the interplay between attitudes, physical activity, and consumption is essential in understanding how lifestyle choices translate into economic actions (Bock et al., 2005; Cui-Jin et al., 2012).

The mediating role of physical activity level has profound implications for sports marketers and policymakers. The statistically significant mediation effect implies that interventions designed to improve physical activity levels could have a ripple effect on increasing sports consumption demand. This is consistent with findings from Chen et al. (2008), who argue that engagement in physical activities can lead to a higher propensity to consume sports goods and services.

Furthermore, the study’s results contribute to the ongoing discourse initiated by Kung et al. (2023) on the impact of sports participation on economic outcomes. By confirming a partial mediation effect, the study underscores the notion that while attitudes toward exercise have a direct impact on consumption, the actual engagement in physical activities is a vital catalyst for realizing this economic potential (Dong and Liu, 2022; Ashdownfranks et al., 2014).

In conclusion, the study advances our understanding by integrating the roles of attitudes and behaviors, thus offering a more nuanced explanation for sports consumption patterns. The evidence suggests that promoting physical exercise not only fosters a healthier populace but can also stimulate economic activity within the sports sector. This dual benefit is particularly pertinent for urban settings like Chengdu, where rapid lifestyle changes present both challenges and opportunities for sports-related businesses and public health initiatives.

## 6 Conclusion

The conclusion of this research underscores that demographic variables significantly differentiate sports consumption demand among urban residents, revealing age and education as influential factors. The analysis confirms a robust, positive relationship between physical exercise attitude and sports consumption demand, mediated by the residents’ physical activity level. This mediation indicates that while attitudes toward exercise influence consumption directly, the translation of these attitudes into actual behavior further amplifies consumption. The findings align with the principles of the Theory of Planned Behavior and the Technology Acceptance Model, which suggest that both intent and behavior significantly shape economic actions. With implications for sports marketers and policymakers, this study highlights the potential of leveraging physical activity to boost sports-related economic activity, emphasizing a strategy that can contribute to both public health and urban economic vitality.

## 7 Limitations and future studies

While this study provides significant insights into the factors influencing sports consumption demand among urban residents, it is not without its limitations. Firstly, the study is geographically restricted to Chengdu, which may limit the generalizability of the findings to other urban or rural settings. Future research could extend this study to diverse geographical locations to verify the applicability of the results across different cultural and economic contexts.

Secondly, the cross-sectional design of this research limits the ability to establish causality between physical exercise attitude, physical activity level, and sports consumption demand. Longitudinal studies are recommended to better understand the dynamics of these relationships over time and to discern any potential causal links.

Additionally, while the mediation model provided valuable insights, the indirect effects identified suggest that other mediating variables might also play significant roles. Future studies could explore additional mediators such as psychological well-being or social influences that could further elucidate the pathway from physical exercise attitudes to sports consumption.

Moreover, significant variances in sports consumption related to age, educational level, overall monthly consumption, and specific sports-related spending highlight potential areas for incorporating moderating variables in future models. This approach could offer a more nuanced analysis of how different segments of the population engage with sports consumption under varying conditions.

Another limitation is the reliance on self-reported data, which can be susceptible to biases such as social desirability or recall biases. Incorporating objective measures of physical activity and consumption, perhaps through tracking devices or financial transaction data, could enhance the accuracy of the findings.

Finally, considering the rapid evolution of digital and mobile health technologies, future research could investigate how these advancements affect physical activity levels and sports consumption habits. This could provide deeper insights into the technological aspects of sports engagement and offer new avenues for promoting physical activity and sports consumption.

Addressing these limitations in future studies will not only strengthen the existing research framework but also expand our understanding of the complex interplay between lifestyle behaviors and economic activities in the sports sector.

## Data Availability

The original contributions presented in the study are included in the article/Supplementary material, further inquiries can be directed to the corresponding author.
